# Letter from Dr. T. B. Gunning

**Published:** 1875-05

**Authors:** 


### ARTICLE IV.

*Editor American Journal Dental Science:*

DEAR SIR—I notice in the report of the second Annual Commencement of the Maryland Dental College, published in the *Cosmos*, April number, the number of matriculants is reported at twenty-four—(24.) While I have no disposition to find fault with the Faculty of that Institution in their efforts at advancing the standard of Dental Education, yet a due regard for facts prompts one to criticise this report, so far at least as these figures are concerned. It is well known to all in Baltimore who are at all familiar with the details of the workings of the Maryland Dental College, that the number of students in actual attendance upon its lectures during the past Winter was seven—(7.) It would seem strange that the other seventeen (17) who matriculated did not attend the lectures. Did they, after paying the fee, see fit to go elsewhere?

Really a college with twenty-four (24) matriculants and seven (7) students in attendance would seem to need looking after by its Board of Regents.

There are some other facts with regard to this College, which place it in an invidious light before the Dental public, but of these hereafter.

Very truly,

The May number of *Cosmos*, corrects the report in the April number, and gives the number of matriculants as 7, instead of 24, as was incorrectly stated.—Ed.

